# Identification and characterization of a new type of inhibitor against the human immunodeficiency virus type-1 nucleocapsid protein

**DOI:** 10.1186/s12977-015-0218-9

**Published:** 2015-11-06

**Authors:** Min-Jung Kim, Seon Hee Kim, Jung Ae Park, Kyung Lee Yu, Soo In Jang, Byung Soo Kim, Eun Soo Lee, Ji Chang You

**Affiliations:** Avixgen Inc., Seoul, 137-701 Korea; National Research Laboratory of Molecular Virology, Department of Pathology, School of Medicine, The Catholic University of Korea, Seoul, 137-701 Korea

**Keywords:** HIV-1, NC inhibitor, Psi RNA dimerization, Noninfectious virus, Gag processing, Core uncoating

## Abstract

**Background:**

The human immunodeficiency virus type-1 (HIV-1) nucleocapsid protein (NC) is an essential and multifunctional protein involved in multiple stages of the viral life cycle such as reverse transcription, integration of proviral DNA, and especially genome RNA packaging. For this reason, it has been considered as an attractive target for the development of new anti-HIV drugs. Although a number of inhibitors of NC have been reported thus far, the search for NC-specific and functional inhibitor(s) with a good antiviral activity continues.

**Results:**

In this study, we report the identification of A1752, a small molecule with inhibitory action against HIV-1 NC, which shows a strong antiviral efficacy and an IC_50_ around 1 μM. A1752 binds directly to HIV-1 NC, thereby inhibiting specific chaperone functions of NC including Psi RNA dimerization and complementary trans-activation response element (cTAR) DNA destabilization, and it also disrupts the proper Gag processing. Further analysis of the mechanisms of action of A1752 also showed that it generates noninfectious viral particles with defects in uncoating and reverse transcription in the infected cells.

**Conclusions:**

These results demonstrate that A1752 is a specific and functional inhibitor of NC with a novel mode of action and good antiviral efficacy. Thus, this agent provides a new type of anti-HIV NC inhibitor candidate for further drug development.

**Electronic supplementary material:**

The online version of this article (doi:10.1186/s12977-015-0218-9) contains supplementary material, which is available to authorized users.

## Background

The human immunodeficiency virus type-1 (HIV-1) nucleocapsid protein (NC) is derived from the Gag polyprotein precursor by the viral protease during viral assembly [1]. It is a small, basic, nucleic acid binding protein with two zinc fingers that are highly conserved among the retroviruses [2–4]. Many previous genetic studies have shown that mutations in the NC result in various phenotypes, which include defects in viral genomic RNA (gRNA) packaging [5–8]. There is also a loss of viral infectivity, abnormality of Gag processing and viral core stability as well as inhibition of viral DNA synthesis in infected cells [9–11]. All these observations are indicative of the undoubted importance of NC in viral replication. The NC plays many essential roles throughout the life cycle of the HIV and is, therefore, considered a new promising and attractive target for the development of new anti-HIV drugs [12, 13].

To date, a number of zinc ejector type of inhibitors targeting the zinc fingers of NC, which are a critical motif for protein function, have been reported. For example, inhibitors like C-nitrosobenzamide (NOBA) [14], disulfide-substituted benzamide (DIBA) [15], 1,2-dithiane-4,5-diol, 1,1-dioxide, cis (Dithiane) [16], azodicarbonamide (ADA) [17], pyridinioalkanoyl thiolester (PATE) [18], thiolcarbamates (TICAs) [19], and S-acyl-2-mercaptobenzamide thioester (SAMT) [20] have been shown to target the zinc ion of the HIV-1 NC and inhibit HIV-1 replication. Subsequently, DIBA, Dithiane, and SAMT have shown similar effects by inducing intermolecular cross-links between cysteins in the zinc fingers of NC in Gag protein. This action causes modification and aggregation of the NC and Gag protein, which results in disturbance of Gag processing [15, 16, 20]. ADA was also reported to induce the modification of NC in viruses and thereby prevent reverse transcription of HIV-1 [17]. However, further development of these types of zinc ejector inhibitors has been mostly limited in part due to either a low or lack of target specificity and cellular toxicity [21, 22]. Recently, there are also other types of NC-zinc ejectors reported such as N,N’-bis(4-ethoxycarbonyl-1,2,3-thiadiazol-5-yl)benzene-1,2-diamine (NV038) and 2-methyl-3-phenyl-2H- [1, 2, 4] thiadiazol-5-ylideneamine (WDO-217), which have not shown covalent bonds in NC [23, 24] unlike the aforementioned inhibitors.

The functional contributions of NC to HIV-1 replication are achieved mostly by its chaperone functions to various forms of viral nucleic acids through specific interactions [25–28]. For this reason, small molecule antagonists, which could inhibit the NC-mediated complementary trans-activation response element (cTAR) DNA destabilization or λ-DNA stretching in vitro, have also been explored [29, 30]. However, the cellular antiviral efficacy of these molecules has shown either quite low [30, 31] or not yet determined [29].

Lately, a series of compounds have been reported to disrupt the binding between NC and synthetic viral nucleic acids without NC zinc ejection, and a few of them exhibited good binding affinity to NC but showed rather modest antiviral activities [32]. In spite of these efforts thus far, the search for new types of small molecule NC inhibitors, which could bind strongly and specifically to NC and inhibit its known functions, thereby effectively suppress viral replication, is still warranted.

Toward the goal, we previously developed a cell-based screening assay system to probe specific interactions between the NC and the viral packaging signal sequence RNA, Psi, element [33] and have screened various chemical libraries that inhibit the interaction. A number of possible inhibitors have been identified, and their antiviral activities have been examined. Here, we report on A1752, a novel small molecule NC inhibitor, which strongly binds the HIV-1 NC thereby inhibiting the chaperone properties of NC and leading to good antiviral activity against the HIV-1.

## Results

### Identification of A1752, a new HIV-1 inhibitor

**Fig. 1:**
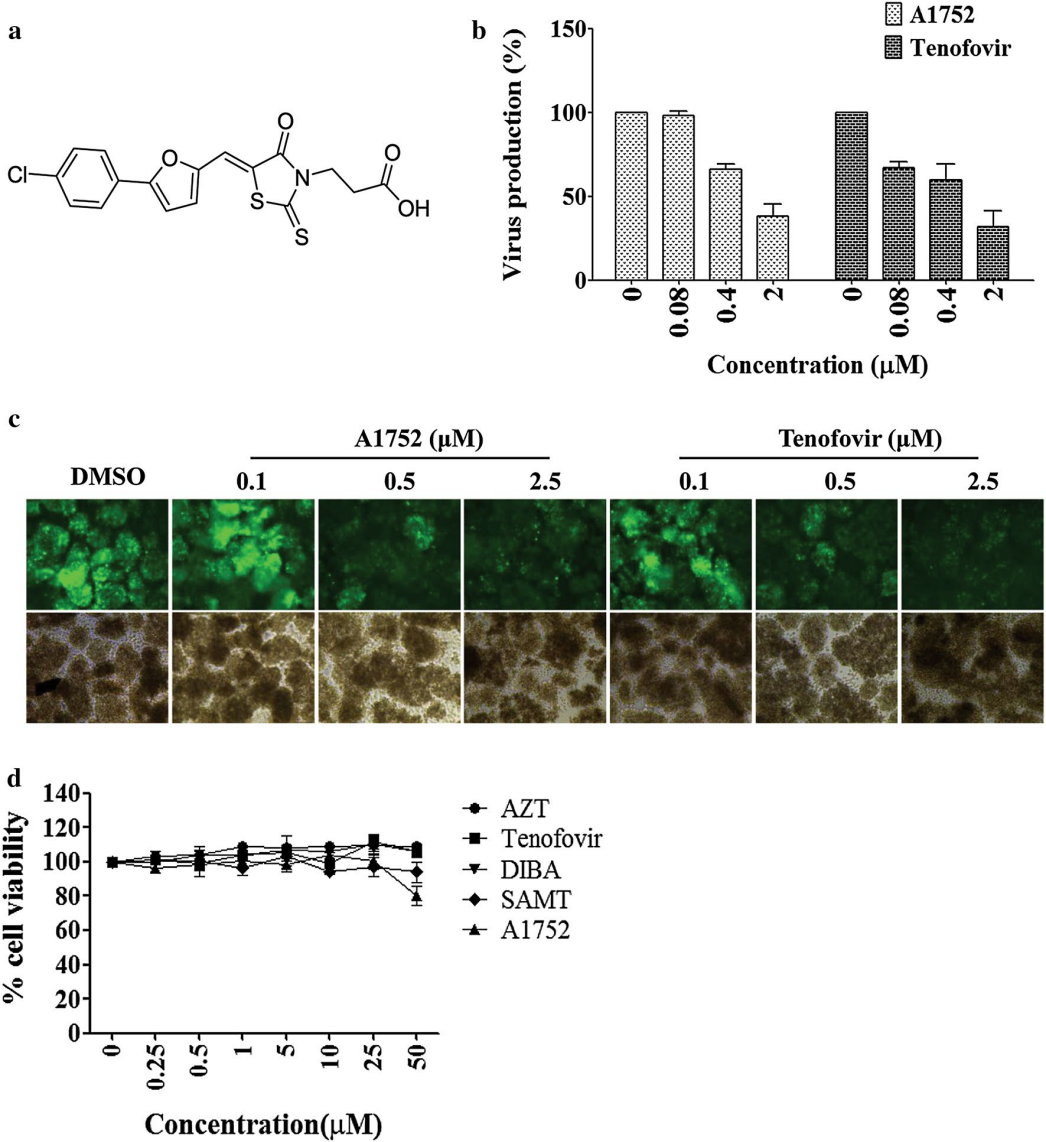
Anti- HIV efficacy of a new small chemical inhibitor A1752. **a** The chemical structure of A1752, (Z)-3-(5-((5-(4-chlorophenyl)furan-2-yl)methylene)-4-oxo-2-thioxothiazolidin-3-yl)propanoic acid. **b** Determination of anti-HIV activity of A1752. Inhibition of HIV-1 production in MT-4 cells infected with a HIV-1 NL4-3/EGFP in the presence of A1752 or a control inhibitor Tenofovir was determined using HIV-1 p24 ELISA. Data are the mean ± SD of triplicate experiments for each concentration. **c** Examination of EGFP expression as a surrogate marker for HIV-1 replication in the infected MT-4 cells treated with the inhibitors in (**b**) was examined on day 3 post infection. **d** Cellular toxicity of the A1752 was measured using a Cell-Titer Glo assay and the percentage cell survival in the presence of each indicated compound compared to that of non-treated cells. Data are the mean ± SD of three separate experiments

To assess the antiviral efficacy of a newly identified small molecule inhibitor designated as A1752 (Fig. 1a) against HIV-1, we used an HIV-1 NL4-3 isolate derivative harboring enhanced green fluorescent protein (NL4-3/EGFP). This allows the viral replication and infection to be easily probed simply by the observation of the EGFP expression in the virus-infected cells [34]. The MT-4 cells were infected with the virus at a 0.05 multiplicity of infection (MOI) and treated with various concentrations of the A1752. The HIV-1 inhibitor Tenofovir was used as the control. After the cells had been incubated for 3 days in which cells were remained mostly viable, the viral production was measured using an enzyme-linked immunosorbent assay (ELISA). The half-maximal inhibitory concentration (IC_50_) of A1752 was determined to be around 1.0–2.0 μM, which is similar to the antiviral efficacy of Tenofovir (Fig. 1b).

Treatment with A1752 decreased dose-dependently the expression of EGFP in the virus-infected cells (Fig. 1c), which nicely supported further the antiviral efficacy of A1752 determined. The observed antiviral effect of A1752 was not mediated by any cellular toxicity since the 50 % cytotoxicity concentration (CC_50_) of A1752 was determined to be much higher than 50 μM (Fig. 1d). Also, we found that A1752 had little effect on the activity of HIV-1 reverse transcriptase (RT) and integrase (IN) (Additional file 1: Figure S1 and Additional file 2: Figure S2), indicating that they are not inhibitory targets of A1752.

### A1752 binds directly to HIV-1 NC protein

**Fig. 2:**
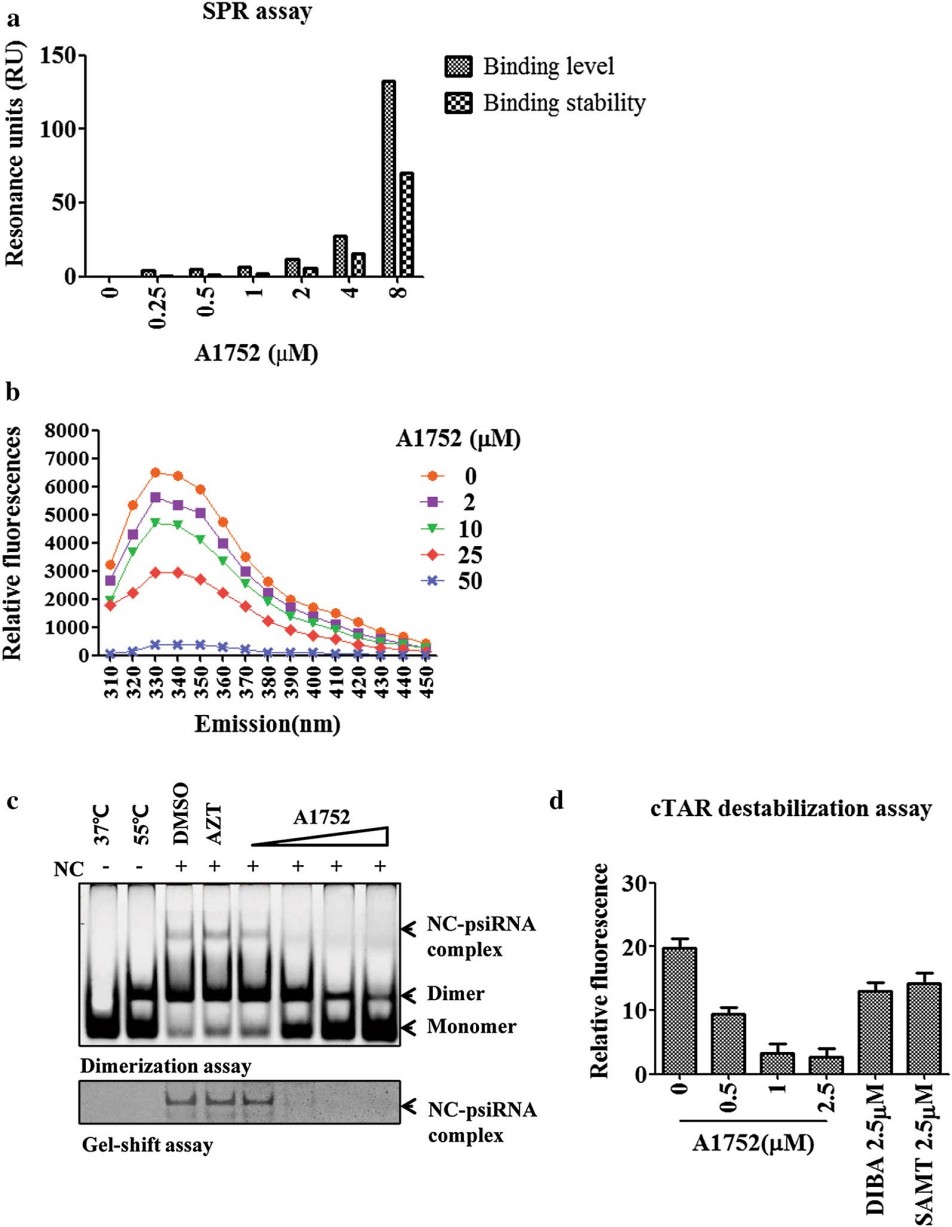
Specific interaction of A1752 with HIV-1 NC and functional inhibition of NC-mediated chaperone activities. **a** The determination of binding affinity of A1752 to NC using SPR assay. **b** Tryptophan fluorescence quenching assay. Shown is intrinsic tryptophan quenching of NC by A1752. NC (5 μM) was used and excitation wavelength was 280 nm and emission was scanned from 310 to 450 nm as shown. **c** Effect of A1752 on NC-mediated HIV-1 Psi RNA dimerization (*Top*) and NC-Psi complex formation as determined by gel-shift assay (*Bottom*). Monomeric and dimeric Psi RNA and Psi RNA-NC complex were probed with a SYBR green staining method for RNA and a SYPRO Ruby staining for protein, respectively. Control bands for monomeric and heat-induced dimeric Psi RNA are also indicated in lane 1 and 2, respectively. NC was incubated with A1752 at increasing molar ratio (1:1, 1:10, 1:25, and 1:50), DMSO and AZT (1:50) as described in “Methods”. **d** Inhibition of NC-induced cTAR destabilization by A1752. NC (1 μM) were preincubated for 10 min with increasing A1752 concentrations or other inhibitors, DIBA and SAMT, as indicated and added to 0.1 μM of doubly-labeled Rh6G-5′-cTAR-3′-DABCYL DNA for 1 h. Fluorescence change was monitored at excitation and emission wavelength of 520 and 560 nm, respectively

To determine in fact the capacity of A1752 to bind specifically to the HIV-1 NC target, we used a surface plasmon resonance (SPR) assay. The titration sensorgram showed that the A1752 bound strongly to the NC dose-dependently (Fig. 2a). The analysis of the binding kinetics result using a 1:1 Langmuir binding model provided by the manufacturer (GE Healthcare) revealed the dissociation constant value (*K*_*D*_) between A1752 and NC to be in the range of 20 nM (Additional file 3: Table S1). In addition, we performed an intrinsic tryptophan fluorescence quenching assay of NC to further examine its interaction with A1752. The result clearly demonstrated that A1752 was able to bind dose-dependently to the HIV-1 NC (Fig. 2b).

### A1752 inhibits NC-mediated dimerization of Psi RNA and cTAR DNA destabilization

Having established the high affinity binding of A1752 to NC protein, we further examined the effects of A1752 on the nucleic acid chaperone function of NC. Firstly, we examined the effects of A1752 on the specific binding of NC to Psi RNA and resulting NC-mediated Psi RNA dimerization. As shown in Fig. 2c, A1752 but not the control compounds inhibited dose-dependently both the NC-induced stable dimerization of HIV-1 Psi RNA and the NC-Psi RNA complex formation. NC is also a known cofactor of HIV-1 RT and mediates its chaperone activities [35]. During the reverse transcription reaction, NC functions in an important role of preventing the self-annealing and -priming of cTAR DNA. This facilitates the synthesis of a proper DNA intermediate at the first-strand transfer step of the reverse transcription. Therefore, we examined the effects of A1752 on NC-mediated cTAR DNA destabilization. The cTAR DNA was double-labeled with 6-carboxyrhodamine (Rh6G) and 4-(4′-dimethylamino phenylazo) benzoic acid (DABCYL), which served as the fluorescence donor and quencher at its 5′- and 3′-ends, respectively, as reported previously [36]. The addition of NC promotes the opening the cTAR stem structure by its chaperone activity. Thus, the fluorescence intensity of the Rh6G-5′-cTAR-3′-DABCYL DNA was increased by treatment with NC alone as expected (Additional file 4: Figure S3). To test inhibitory effect of A1752, the NC was pretreated with A1752 as well as the previously reported NC zinc finger targeting inhibitors, DIBA or SAMT. The fluorescence intensities were decreased concentration-dependently by A1752, indicating that it inhibited the cTAR destabilization activity of NC. Interestingly, both DIBA and SAMT showed a little effect on the NC-mediated cTAR destabilization under the same condition (Fig. 2d). A similar lack of inhibition was also observed in the NC-mediated Psi RNA dimerization assay (data not shown). These results together suggest further that A1752 is a bona-fide functional inhibitor that acts by specifically binding to NC and suppressing the NC-associated chaperone functions.

### Treatment with A1752 produces noninfectious HIV-1

**Fig. 3:**
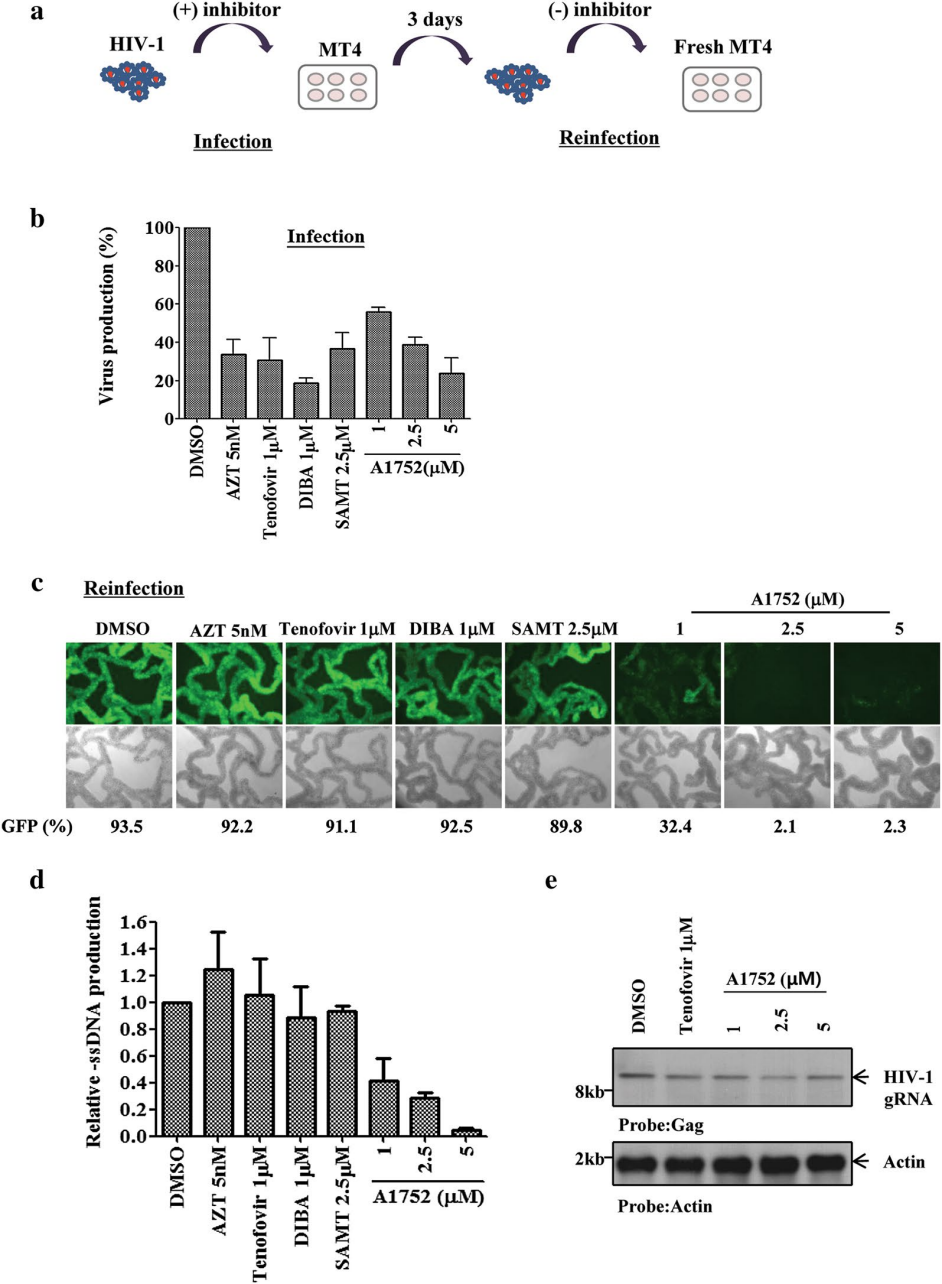
Generation of noninfectious virus by A1752. **a** A schematic representation of a reinfection experiment to examine infectivity of virus produced. **b** Comparison of antiviral activity of A1752 with other anti-HIV inhibitors. MT-4 cells were infected with HIV-1 NL4-3/EGFP virus in the presence of the indicated inhibitors. Viral production was determined using HIV-1 p24 ELISA. AZT, Tenofovir, DIBA, and SAMT were also used for comparison. **c** Determination of infectivity (Reinfection assay) of the viruses obtained in **b**. Fresh MT-4 cells were infected with the same amount (2.5 ng in p24) of purified viral particles produced from the infection assay in (**b**) in the absence of any inhibitors. Then, the GFP expressing cells were quantified using FACS analysis 48 h after the infection. **d** The viral (-)ssDNA, an early RT product, produced for 6 h after infection with viruses of each case from (**b**) was determined using qPCR. Data are the mean ± SEM of three separate experiments. **e** Determination of HIV-1 gRNA in MT-4 cells infected with an equal amount (30 ng in p24) of the virus particles produced from (**b**). At 3 h after infection, total cellular RNA was analyzed using northern-blot for detection of gRNA in the infected MT-4 cells. Gag-specific probe was for HIV-1 gRNA and an actin probe was used as a loading control

To understand molecular mechanisms of the antiviral activity of A1752, we examined the infectivity of viral particles generated after treatment with A1752 using a reinfection assay as described schematically in Fig. 3a. Firstly, the MT-4 cells were infected with the HIV-1 NL4-3/EGFP virus and then treated with A1752 or the other control compounds. Similar to the results illustrated in Fig. 1b, viral production was again significantly inhibited by A1752 as all the other control inhibitors used (Fig. 3b). Next, we examined the infectivity of the resulting viruses produced. This was achieved by infecting fresh MT-4 cells with the same amount of purified and quantified virus particles released from the infection in each case. There were no further chemical inhibitors added or carry-over conditions as described in the “Methods”. As shown in Fig. 3c, all the viruses generated in the presence of the other inhibitors including azidothymidine (AZT), Tenofovir, DIBA, and SAMT showed an equal level of infection like the DMSO positive control. Surprisingly, however, infection of the viruses generated from the A1752-treated cells was attenuated sharply and A1752 dose-dependently (Fig. 3c), indicating the production of non-infectious virus by the A1752.

### Virions generated in the presence of A1752 are defective in synthesis of the viral early RT product in the virus-infected cells

To further characterize the inhibition and loss of viral infectivity induced by the A1752, we examined the synthesis of RT products in the virion-infected cells. We measured the minus strand strong-stop (-)ssDNA, an early viral RT product, using the quantitative real-time polymerase chain reaction (qPCR) method. Notably, the production of the (-)ssDNA was significantly decreased in cells infected with virion generated in the presence of A1752 (Fig. 3d). The synthesis of the (-)ssDNA product was suppressed up to 60 % at 1 μM and nearly 90 % at 5 μM, even though the viral RNA template was detected at a similar level in the infected cells (Fig. 3e), This result indicated that RT reaction following the infection was non-productive and accounts in part for the appearance of the A1752-induced defective and non-infectious virus phenotype. It is noteworthy that this phenomenon was not caused by the inhibition of RT activity by A1752 as it was determined that the HIV-1 RT reverse transcriptase was not affected by A1752. In addition, we observed that at a higher concentration of around 20 μM of A1752, the HIV-1 gRNA packaging was inhibited to some extent (Additional file 5: Figure S4), suggesting that higher concentrations of this inhibitor might also affect gRNA packaging mediated by NC during viral RNA assembly.

### A1752 produce non-infectious viruses in a proviral DNA and lentiviral vector transfection system

**Fig. 4:**
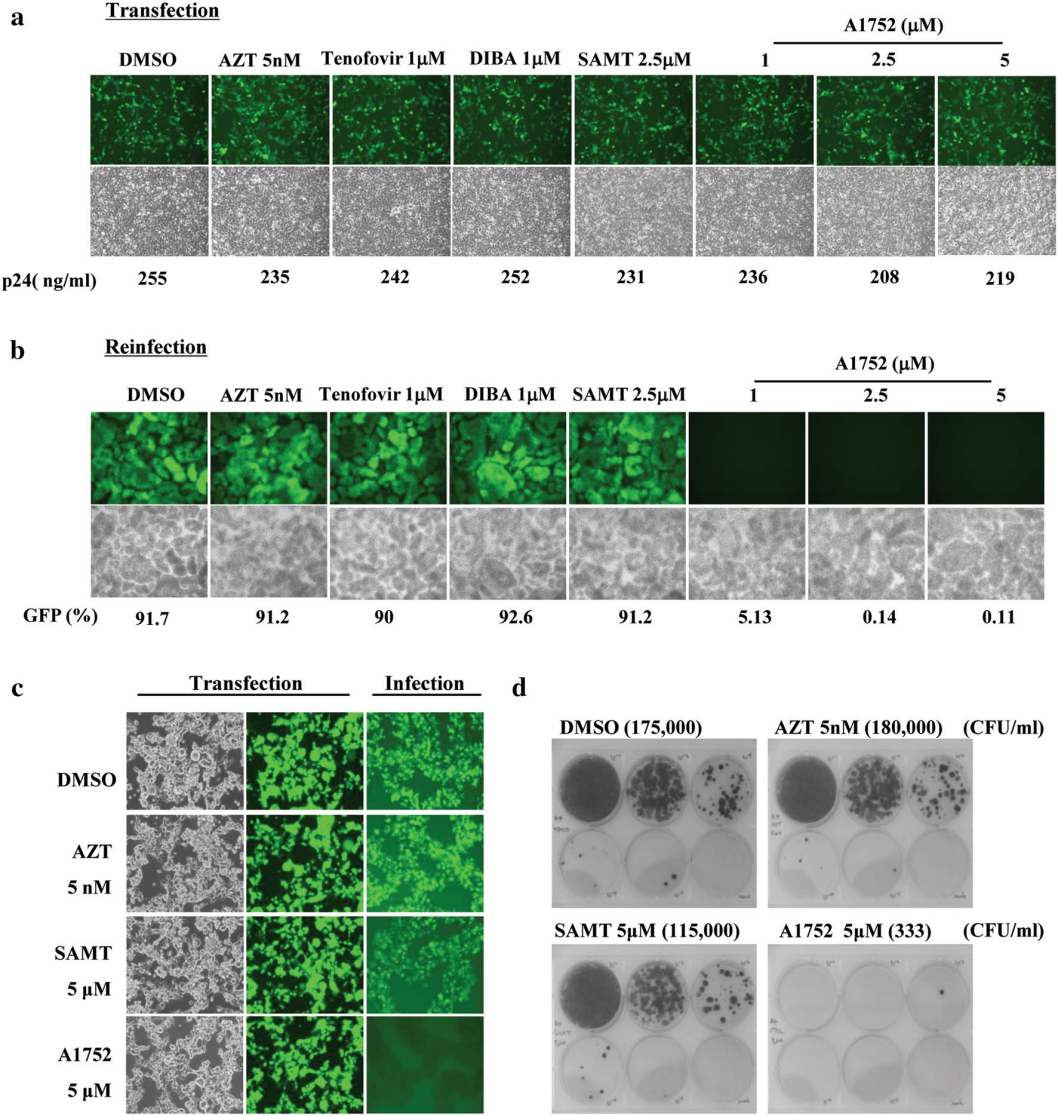
Effect of A1752 on infectivity of viruses generated by proviral DNA transfection and lentiviral system. The 293FT cells were transfected with a pNL4-3EGFP HIV-1-proviral DNA in the presence of the indicated inhibitors. **a** Post-transfection 48 h, EGFP expression within cells and viral production were analyzed, respectively. **b** An equal amount (2.5 ng in p24) of the virions produced in each case in (**a**) was taken to infect to MT-4 cells as described in Fig. 3, and the infection was analyzed using both a fluorescence microscopic observation of EGFP expression and quantitative FACS analysis (% of GFP) 48 h after infection. **c** 293FT cells were transfected with a pLenti/EGFP transgene plasmid and packaging plasmids (pLP1, pLP2, and pLP/VSVG) in the presence of the inhibitors indicated (middle column) The same amounts (10 ng in p24) of lentiviral vectors produced under the conditions were subjected to infection to fresh MT-4 cells and followed by determination of level of EGFP expression (the *last column*), and **d** HT1080 colony assay for the determination of the lentiviral vector infectivity. The colony forming units (CFU) were compensated with p24 ELISA values for the virus particles used in each case. Data are the mean ± SEM of three separate experiments

The non-infectious virus phenotype observed in the reinfection assay suggests that the antiviral activity of A1752 most likely occurs in the late stage of the virus life cycle, which includes the viral packaging and assembly stage. To further confirm this, we first examined the infectivity of the virus generated using a transfection system of the HIV-1 proviral DNA, which reflects only the late stages of the HIV-1 life cycle following viral integration. The 293FT cells were transfected with a pNL43/EGFP proviral DNA followed by treatment with A1752, and then the transfected cells and viral supernatant were analyzed 48 h after transfection. The results showed that A1752 had no influenced on the viral gene expression since the same level of EGFP expression was observed in all the test cases (Fig. 4a). In addition, the viral translation and budding for virus production was also unaffected. This was verified by the p24 ELISA measurements showing that A1752 had no inhibitory effect on the production and release of virus particles (bottom of Fig. 4a). A similar result was also obtained with all the other control HIV-1 inhibitors including AZT, Tenofovir, DIBA, and SAMT.

Next, to test the infectivity of the viruses released from the transfected cells in each case, fresh MT-4 cells were infected again with an equal amount of the viral particles produced. The infections were successfully observed in cases with all the other control compounds including AZT, Tenofovir, DIBA, SAMT, and DMSO. However, the viral infectivity was completely attenuated at an inhibition level of over 95 % with 1 μM of A1752, despite the normal level of virus production (Fig. 4a, b). This result suggests clearly that the viral particles produced in the presence of A1752 were non-infectious as seen in the MT-4 cell infection system.

To further confirm the generation of non-infectious viruses, we also examined the effect of A1752 on the viruses generated using a lentiviral vector system, which uses an heterologous viral entry glycoprotein VSV-G rather than that of HIV-1. This system permits only a single round of infection and, for this reason, is an excellent system to evaluate the effects of A1752 on only the viral assembly and maturation step. The results also revealed no observable differences in the expression of the green fluorescence protein (GFP) following transfection with the lentiviral vector with or without the inhibitors tested, showing an equal transfection efficiency and production of lentiviruses (Fig. 4c). However, the infectivity of the lentiviruses generated in the presence of A1752 but not AZT or SAMT, was significantly decreased in both the GFP-positive infected cell count and a separate cell colony assay used for lentivirus titer determination (Fig. 4c, d). These results clearly demonstrate further that treatment with the NC inhibitor A1752 leads to the release of non-infectious defective viruses. This also confirms that that the inhibitory action of the A1752 occurs in the late phase of the virus life cycle such as viral packaging and maturation and thus rules out further a possibility of A1752 acting as an entry inhibitor of HIV-1.

### Time-of-addition (TOA) assay: the antiviral effect of A1752 occurs in the late phase of HIV-1 replication

**Fig. 5:**
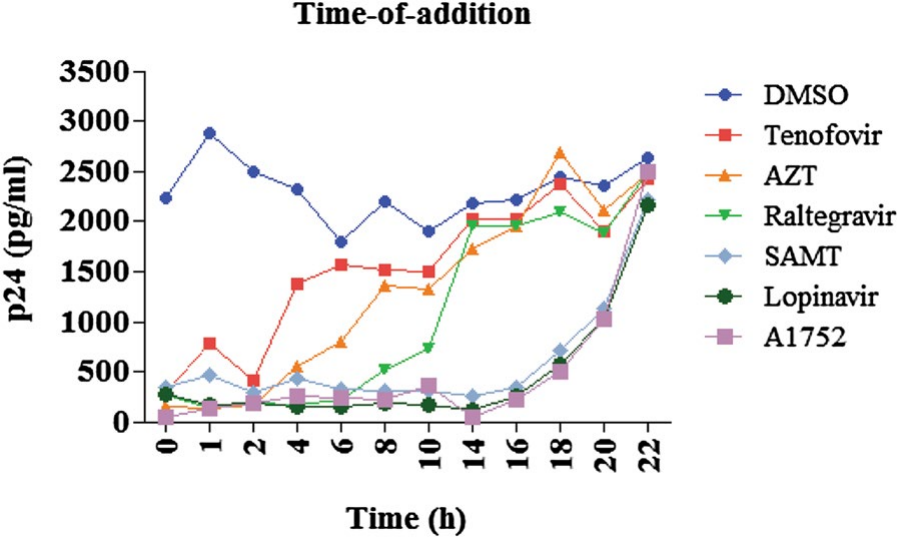
Time of addition assay. The MT-4 cell were treated with the indicated inhibitors every 2 h for up to 24 h after virus infection and then virus production was determined at the indicated times using HIV-1 p24 ELISA as described in “Methods”

To further verify the point at which A1752 specifically acts in the viral life cycle, we also performed a TOA assay as described previously [37]. Following infection, the MT-4 cells were treated at various time points with the indicated control HIV-1 inhibitors. The inhibitors used for the analysis were AZT, Tenofovir, and Raltegravir, representing the HIV-1 life cycle early phase inhibitors, while SAMT and Lopinavir were the late phase inhibitors. The viral supernatants were collected, and viral production was analyzed for 24 h after the infection (Fig. 5). While viruses were produced continuously over the 24 h period in the DMSO control, virus production was suppressed within 2 h by treatment of AZT and Tenofovir, 6 h by Raltegravir, and 14 h by SAMT and Lopinavir, respectively. This was expected and showed the reliability of the experimental condition as previously reported [37]. Under these conditions, the A1752 suppressed the viral production at around 14 h similar to Lopinavir and SAMT, which confirmed further that A1752 functions as a late-phase inhibitor of HIV-1. This also correlated well with the target point of its antiviral activity where the NC is known to function most importantly, mediating the production of infectious viruses.

### A1752 inhibits the proper processing of Gag proteins

**Fig. 6:**
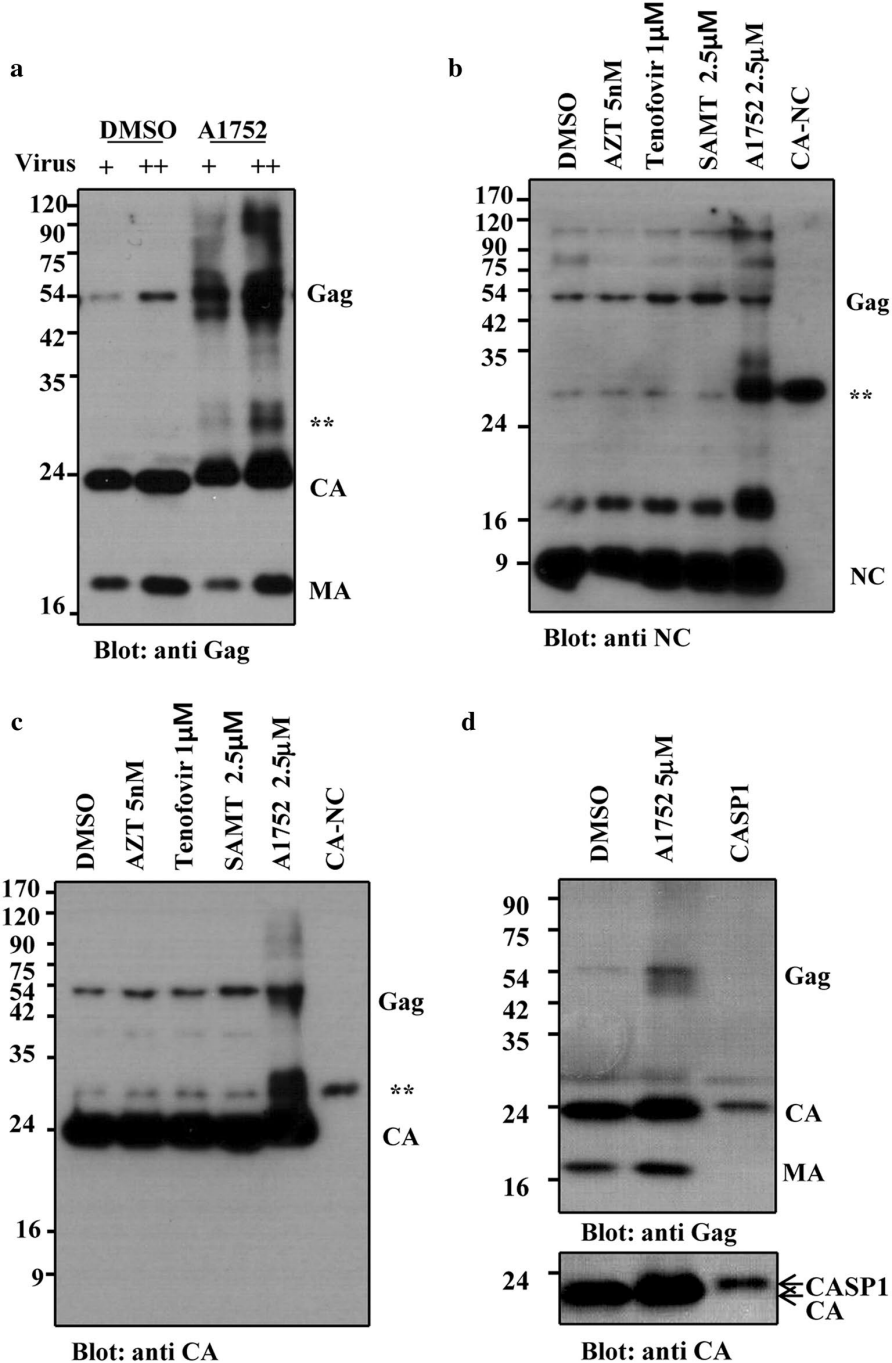
A1752 generates aberrant Gag protein processing. The virus particles produced from MT-4 cells in the presence of A1752 (**a**, **d**), and from 293FT cells transfected with HIV-1 proviral plasmid and treated with the indicated inhibitors (**b**–**c**) were analyzed using western blot assay. The membranes were probed with either anti-Gag (**a**, **d**), −NC (**b**) or −CA (**c**, **d**) antibodies as indicated, respectively. A CA-NC and CASP1 form (25 KD) of Gag protein expressed in *E. coli* were used as a control. *Double asterisk* indicates a specific major protein band (30 kD) generated by A1752

It has been shown by many genetic studies that one of the phenotypes generated by mutations in NC is the failure of viral particles to attain full maturation due to the inhibition of proper gRNA dimerization and Gag polyprotein processing [38]. This eventually results in a loss of viral infectivity. It was also reported previously that NC inhibitors like DIBA and SAMT at high concentrations generated unprocessed Gag proteins, which accumulated in virion [20, 39]. Therefore, we investigated the effects of A1752 on the processing of Gag proteins in virion. Interestingly, a protein band of about 30 kD was distinctively and specifically detected with A1752 treatment (Fig. 6a). Moreover, the level of unprocessed Gag increased notably, Capsid (CA) was slightly shifted, and molecules higher than 75 kD were also accumulated in the virion dose-dependently (Fig. 6a). Particularly, we estimated that the 30 kD protein band might be the size of CA-NC and might have been produced by the incomplete cleavage between CA and NC during the processing of the Gag protein. To clarify this observation, we analyzed the virion proteins in a parallel comparison with bacterial cell lysates expressing the CA-NC fusion protein and identified the 30 kD protein band as a form of CA-NC, which was indeed probed equally with NC and CA antibodies (Fig. 6b, c). In addition, the CA band slightly shifted in Fig. 6a was appeared to be an unprocessed form of CA and SP1. To demonstrate this further, we also analysed the virion generated with A1752 treatment in comparison with bacterial cell lysates expressing a CASP1 fusion protein (Fig. 6d), confirming further that the shifted band is undoubtedly the CASP1 form. These suggest that the generation of the abnormal protein might be due to interference in the processing of the junctions between CA and NC caused by the binding of A1752 to NC in the Gag protein.

### A1752 defers uncoating of HIV-1 core in infected cells

**Fig. 7:**
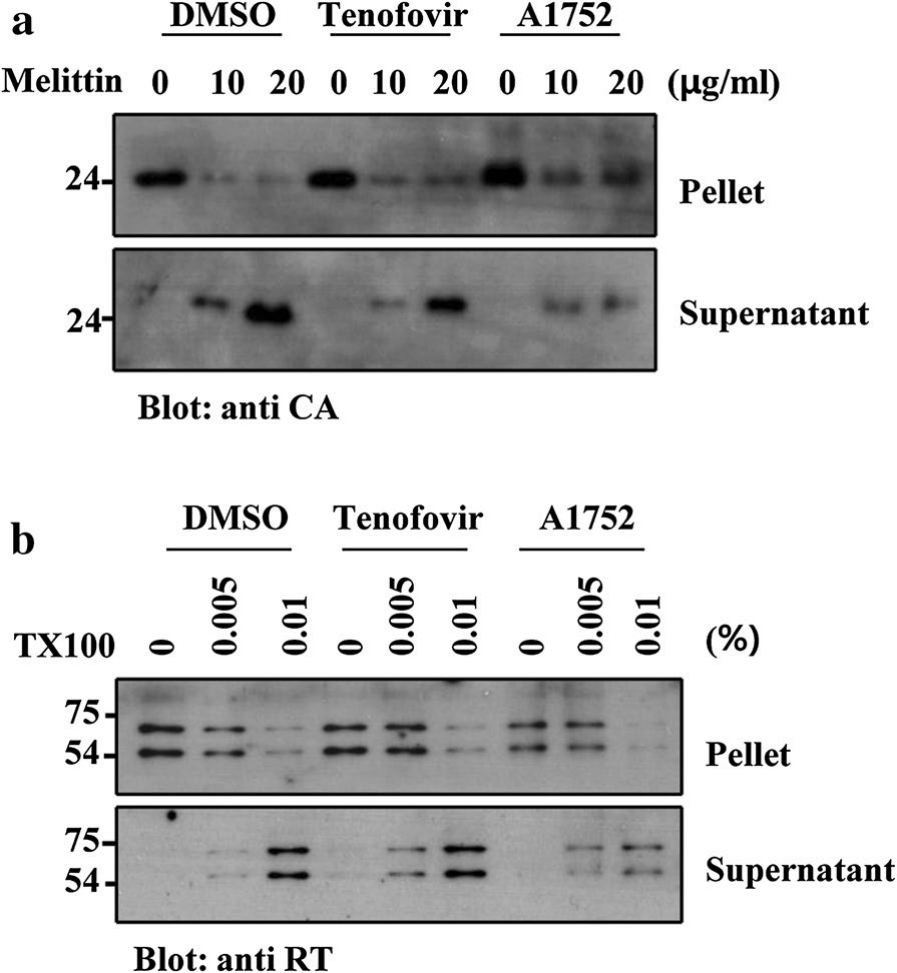
A1752 induces abnormal HIV-1 core stability. **a**, **b** The virus particles produced from HIV-1 proviral plasmid-transfected 293FT cells were treated with A1752 and permeabilized either by Melittin (**a**) or Triton X-100 (**b**) at room temperature for 10 min and then exposed to a 37 °C for 30 min to disassemble the HIV-1 core structure. The resulting viruses were fractionated to a pellet and supernatant by centrifugation as described in “Methods”, and subjected to western blot analysis with anti-CA (**a**) or anti-RT (**b**) antibodies

The precise processing of the Gag protein is required for proper formation of HIV-1 cores, which is essential for a productive RT reaction for viral infectivity [40]. Therefore, we investigated whether the inhibition of the Gag processing by A1752 could also induce an immature or abnormal HIV-1 core, which would inhibit the reverse transcription as observed in Fig. 3d. To examine this possibility, we analyzed the stability of the HIV-1 virion core produced in the presence of A1752 as reported previously [41]. It has been reported that the immature core is hyper-stable compared to the normal core and results in a slower uncoating rate [42], which has also been associated with the impaired replication phenotype. To examine the core integrity, we first obtained viruses from 293FT cells transfected with the HIV-1-proviral DNA and also treated with A1752. An equivalent amount of the viruses were permeabilized with Melittin or Triton X-100 and then incubated at 37 °C for core disassembly and centrifuged at 28,500×*g* for 1 h 30 min. The resulting pellet and the supernatant fraction were analyzed using a western blot to probe the CA in the HIV-1 core and free CA protein, respectively. Exposure of the virions to increasing concentrations of Melittin (10–20 μg/mL), or Triton X-100 (0.005–0.01 %), released the HIV-1 CA and RT proteins from the disassembled core, thereby causing them to appear more in the supernatant fraction compared to the simultaneously analyzed pellet fraction (Fig. 7 and Additional file 6: Figure S5). In contrast to the DMSO and Tenofovir control, treatment with A1752 caused the CA and RT proteins to be retained considerably more in the pellet fraction compared to the supernatant fraction under the same permeabilization conditions. This indicates that the cores of the virion modified by the A1752 are hyper-stable compared to the others. These data suggest that the A1752 also affects the stability of the HIV-1 core as induced by the abnormal or immature core resulting from the improper Gag processing. Collectively, the results suggests that the novel phenotype of the noninfectious virus production generated by A1752 would most likely be attributable all to the specific interaction of A1752 with NC, which inhibited the NC chaperone function and led to the abnormal processing of the Gag protein in the virion generated.

## Discussion

The HIV/acquired immune deficiency syndrome (AIDS) pandemic remains a global health problem. The anti-HIV drugs currently developed have been effective in controlling the progression of severe infection. However, the emergence of drug-resistant strains requires the urgent identification of new types of inhibitors with mechanisms of inhibition that differ from the existing drugs [43, 44]. The HIV-1 NC has been suggested to be a prime target for the development of new types of anti-HIV/AIDS inhibitors. NC is an essential protein required in many steps of viral replication and mutations in NC causes various abnormalities in the viruses, thereby decreasing its infectivity.

In this study, we identified a new NC-inhibitor, A1752, which showed good antiviral efficacy, and binds directly to HIV-1 NC with a strong affinity in the nM range of Kd (Fig. 2a). In addition, it effectively inhibited the nucleic chaperone functions of NC. The NC is required for the recognition of the Psi sequence in the viral gRNA, which is followed by dimerization and packaging of gRNA during viral assembly [45]. Our results showed that A1752 specifically and dose-dependently inhibited the NC-induced Psi RNA dimerization by interfering with the specific interaction between the Psi RNA and NC (Fig. 2c). In addition, we observed that A1752 inhibited the NC-mediated destabilization of cTAR DNA hairpin doubly-labeled at ends with a fluorophore/quencher pair (Fig. 2d). Moreover, treatment with A1752 produced viral particles that completely lost their infectivity (Figs. 3c, 4). Furthermore, this effect was far more efficient than some NC zinc finger inhibitors, which were previously reported to induce the loss of viral infectivity but required much higher doses than ours [20]. In addition, we also observed that the A1752 disrupted the proper processing of Gag. Specifically, the cleavage of the CA and NC precursor (CA-NC) was disrupted (Fig. 6). These results strongly suggest that one of the underlining mechanisms of the generation of the non-infectious phenotype by A1752 might be associated with the improper Gag processing of viral protease. This improper processing may be as a result of the modification of the NC domain induced by the binding of A1752 during viral maturation. Previously, it was also shown that some of the known zinc ejector NC inhibitors induced the modification of NC and cross-linking of Gag protein [20, 39]. For example, SAMT inhibits the Gag processing although this effect was observed at a rather high concentration of 100 μM and was barely detected at the level of its IC_50_ (Fig. 6). In contrast, A1752 is much more efficient in this phenomenon, indicating that it is a highly effective inhibitor of NC.

HIV-1 maturation, particularly normal formation and processing of the viral core, is very critical for optimal viral infectivity [46]. The generation of an immature/aberrant HIV-1 core is a phenotype that is frequently observed with NC mutants [9–11] and more recent evidence strongly suggest that the proper uncoating of the virus is an essential step at the early stage of infection of target cells [47]. The A1752 appears to have induced an increase in the unprocessed CA-NC precursor as seen in Fig. 6, which might have caused incomplete viral maturation resulting in a deficiency in the functionality of the HIV-1 core formation [48]. Therefore, we further examined the viral core abnormality induced by the A1752 and found indeed that it was much more refractory compared with the wild types in releasing the RT proteins in core (Fig. 7). This would have led to the failure of the reverse transcription as shown by our detection of the significant suppression of the synthesis of early RT products (-ss)DNA (Fig. 3d). Therefore, it could be deduced that the modification of the core stability induced by the A1752 might also have inhibited the normal uncoating process in the modified viral infected cells. Furthermore, based on the inhibition of the NC-mediated cTAR destabilization by the A1752 (Fig. 2d), a similar inhibition of the chaperone activities of the NC by the A1752 could also have contributed to defects in the RT process post infection.

## Conclusions

In summary, we have identified A1752, a new type of NC inhibitor, which specifically binds to the relevant target and exhibits good antiviral activity. The A1752 strongly binds to NC and thereby interferes with the chaperone functions of NC as well as the Gag processing. Consequently, this action produces non-infectious and abnormal viruses that are defective in uncoating and viral reverse transcription in cells following infection. Therefore, these results suggest that these unique properties of A1752 support the proposition that it is a functional NC inhibitor and could serve as an excellent novel candidate molecule for the development of a new anti-HIV drug.

## Methods

### Infection, transfection, and reinfection

The MT4 and 293FT cells were maintained in RPMI and DMEM, respectively, and the media contained 10 % FBS (Hyclone, Logan, UT), penicillin, and streptomycin sulfate (GIBCO, Carlsbad, CA). The MT4 cells were infected with the NL4-3 isolate-derived HIV-1, which had the *nef* gene replaced with EGFP and were treated simultaneously with the inhibitors as indicated. The 293FT cells were typically transfected with 1 μg the NL43/EGFP-proviral DNA using lipofectamine2000 (Invitrogen, Carlsbad, CA). Following a 6-h transfection, the media were changed and cells were treated with the test compounds. Post infection day 3 or post transfection 36 h, the viral supernatants were collected for further analysis and the remaining cells were analyzed for GFP expression. For the reinfection assay, the viral supernatant produced in the presence of the compounds was first centrifuged at 900×*g* for 10 min, filtered through a 0.45-μm filter to remove the cell debris. The viruses were then further purified and concentrated by centrifugation at 28,500×*g* for 3 h to remove any remaining inhibitors used in infection assay. The resulting pellet was resuspended in serum-free fresh RPMI to yield virus stocks. An equivalent amount (typically 2.5 ng) of virion was used to re-infect fresh MT4 cells. Post infection at 48 h, the expression of GFP was examined followed by a FACS analysis for quantification.

### HIV-1 p24 ELISA assay

The amounts of virus particles of each sample were determined using an HIV-1 p24 ELISA kit as recommended by the manufacturer’s instruction (Advanced Bioscience Laboratories, Rockville, MD), which uses monoclonal antibodies against p24 epitopes for the detection of p24 antigen.

### Lentivirus preparation and HT108 cell colony assay

The pLenti/EGFP transgene vector and packaging plasmids pLP1, pLP2, and pLP/VSVG plasmids encoding the viral proteins Gag-Pol, Rev, and VSV-G, respectively, were prepared and used for transfection as described previously [49]. The infectivity of the lentivirus generated with and without the inhibitors was determined using a colony assay following the manufacturer’s instructions (Invitrogen, Carlsbad, CA). Briefly, 1 × 10^5^ HT1080 cells were first placed in a 35 mm dish. On the following day, the lentiviral supernatants were serially diluted tenfold five times in a total volume of 1 ml, and added to each well to attain a 6 μg/mL final concentration of polybrene (Sigma, St. Louis, MO). After incubation at 37 °C overnight, the media were replaced with 2 mL of DMEM. After a day, the media were changed again to fresh DMEM containing blasticidin (5 μg/mL final concentration, Sigma) and the cells were incubated further at 37 °C for 14 days with media changes performed every other day. After 14 days of selection, each well was washed twice with 1 mL PBS and incubated with 1 mL of crystal violet solution (1 % crystal violet, Sigma, 10 % ethanol) for 10 min at room temperature. Then the cells were washed with 1 mL PBS four times to remove the excess crystal violet, and the stained colonies were counted for viral titer determination.

### Cell cytotoxicity assay

The cytotoxicity was measured using an ATP-based assay. For the determination of the CC_50_ of the compounds, MT4 cells (1 × 10^4^ cells) were seeded in a white 96-well plate in 100 µL volume with compounds (0–50 μM). After 5 days, the plate was treated with 25 μL of CellTiter-Glo reagent (Promega, Fitchburg, WI) per well, mixed for 2 min on shaker to induce cell lysis, and then further incubated at room temperature for 10 min and subjected to luminescence recording on a spectraMax luminescence microplate reader (Molecular Device) according to the manufacturer’s instructions.

### SPR and tryptophan fluorescence quenching assay

For the SPR assay, the NC protein (GenScript, Piscataway, NJ) was immobilized to a CM5 chip contained in a 10 mM sodium acetate buffer (pH 5.5) solution. The test compounds were diluted in 5 % DMSO and allowed to flow on chip in PBS running buffer at the rate of 30 μL per min for 120 s. The affinity was measured using a Biacore T100 instrument and the data evaluation was processed to acquire all the binding strength and kinetic parameters with the analysis program provided by the manufacturer (GE Healthcare, USA) [50, 51]. The tryptophan quenching of NC (5 μM) was performed in a buffer containing 10 mM sodium phosphate and 10 % glycerol with or without A1752. The decrease in fluorescence was measured at excitation and emission wavelengths of 280 and 340 nm, respectively, using a spectraMax Gemini Em fluorescence microplate reader (Molecular Device).

### Psi RNA dimerization and gel shift assay

The pcDNA/psi/EGFP plasmid which contain a T7 promoter directly upstream of the HIV-1 Psi sequence (895–1019) [52] was linearized by a *BamH*I as a template for the transcription. The Psi RNA molecules were prepared in vitro using the RiboMAX™ large scale RNA production systems SP6 and T7 (Promega, Fitchburg, WI) following the manufacturer’s protocol. A total of 5 μM of NC was incubated with A1752 or AZT at an increasing molar ratio of A1752 (1:1, 1:10, 1:25, and 1:50) or AZT (1:50) to NC at room temperature for 30 min. Then, 2 μM of Psi RNA was denatured for 10 min at 105 °C and then chilled on ice for 5 min. The denatured RNA was then incubated with the NC-compound mixture in the following order, 10 μL of NC (5 μM)-compound mixture, 4 μL of 5 × NC buffer containing 100 mM Tris–Cl (pH 7.5), 250 mM NaCl, 25 mM dithiothreitol, 1 mM MgCl_2_, and 6 μL of denatured RNA (3 μM). The mixtures were incubated for 30 min at room temperature. At the end of the incubation, the mixtures were loaded on a pre-run 8 % non-denaturing polyacrylamide gel and electrophoresed at 100 V for 40 min in TBE buffer. After electrophoresis, the RNA was visualized by staining with SYBR^®^ Green I nucleic acid gel-stain solution (Molecular Probes. Carlsbad, CA) The RNA-binding protein was visualized by staining with SYPRO^®^ Ruby EMSA protein gel-stain solution (Molecular Probes. Carlsbad, CA).

### cTAR DNA destabilization

The doubly-labeled cTAR DNA oligonucleotides (55 nts) were synthesized at a 0.2 μmol scale and purified by the manufacturer (Bioneer Inc., Daejeon, Korea). The labeling dyes were Rh6G and DABCYL at the 5′ and 3′ ends of the cTAR DNA, respectively. All experiments were performed at room temperature in 25 mM Tris–HCl (pH 7.5), 30 mM NaCl, and 0.2 mM MgCl_2_, and the NC was incubated with labeled cTAR DNA at a molar ratio of 10:1. The fluorescence was recorded on a spectraMax fluorescence microplate reader (Molecular Device) and the excitation and emission wavelengths were 520 and 560 nm, respectively.

### Analysis of viral DNA in infected cells

The MT4 cells (1 × 10^5^) were with infected HIV-1 corresponding to 20 ng of HIV-1 p24, unless specified otherwise, and then harvested after 6 h. The total cellular DNA was extracted using the DNeasy blood and tissue kit (Qiagen, Hilden, Germany) according to the manufacturer’s instruction and analyzed by qPCR using the following HIV-1- specific oligonucleotides, Forward (5′-CAAGTAGTGTGTGCCCGTCTGTT-3′), Reverse (5′-CTG CTAGAGATTTTTCCACACTGAC-3′).

### Viral RNA analysis

The total RNA after viral infection was extracted with Trizol reagent (Invitrogen, Carlsbad, CA) according to the manufacturer’s instructions. The viral RNA was analyzed using a northern blot assay. A total of 3 μg of RNA was denatured at 68 °C in sample buffer containing 6.5 % formaldehyde, 50 % formamide, 1X MOPS, 5 % glycerol, and 0.04 % bromophenol blue for 15 min and separated on a 0.8 % agarose gel containing 2.2 M formaldehyde. This was then transferred to nylon membrane (Roche, Indianapolis, IN). After transfer, the RNA was fixed to the membrane by UV cross-linking. For hybridization, a digoxigenin (DIG)-labeled riboprobe system was used. The riboprobes which corresponded to the Gag (790–1285 nt) sequences of pNL43GFP were synthesized by in vitro transcription using T7 polymerase according to the instruction of the Dig Northern Starter Kit (Roche, Indianapolis, IN), and the actin probe provided in the kit was used. The hybridization and detection were performed according to the manufacturer’s instructions.

### HIV-1 Core uncoating assay

An equivalent amount of viruses were pemeabilized with the reagents such as Melittin (Sigma) or Triton X-100 and then exposed to heat for 30 min to disassemble the HIV-1 core structure as described previously [41]. The resulting viruses were centrifuged for 1 h 30 min at 28,500×*g*. The resulting pellet and supernatant fractions were analyzed using western blot.

### Western blot analysis

An equivalent amount virion was heat-denatured in the sample buffer containing 8 % SDS, 250 mM Tris–HCl (pH 6.8), 40 % glycerol, 0.02 % bromophenol blue, and 5 % β-mercaptoethanol (95 °C) for 10 min and then analyzed by SDS-PAGE (12 %). After transfer the proteins to the membrane it was probed with anti-p55 (1:10,000, Thermo Scientific, Pittsburgh, PA), anti-p24 (1:10,000, Abcam, Cambridge, MA), anti-NC (1:5000, a gift from Dr. Robert J. Gorelick at the NCI-Frederick Cancer Research), and anti-RT antibodies (1:10,000, Abcam, Cambridge, MA).

### Time of addition (TOA) assay

The TOA assay was performed as described previously [37]. Briefly, MT4 cells were infected with the NL43-derived HIV-1 bearing GFP (0.5 MOI) and treated with the test compounds at concentrations corresponding to 10–100 fold of the IC_50_ of each compound at predetermined times. Post infection (24 h), the cells were analyzed to detect the GFP content and the viral supernatants for the virus production were titrated using a p24 ELISA.

### HIV-1 reverse transcriptase assay

The RT assays were performed using a non-radioactive fluorometric method [53]. The poly(A) substrates, oligo(dT) primers and PicoGreen fluorophore were provided by the EnzChek reverse transcriptase assay kit (Invitrogen, Carlsbad, CA). After hybridization, the primers were elongated to long RNA-DNA heteroduplexes in the presence of the RT (3 units, Abcam) with or without the test compounds, and the formation of the heteroduplexes was correlated with the RT activity. Finally, the PicoGreen fluorophore incorporating into the RNA-DNA duplexes was added, and the activity of the RT was measured using a fluorometer. The concentration of the test compound was 0.3–100 μM.

### HIV-1 integrase assay

The integrase assays were colorimetrically performed using a HIV-1 integrase assay kit (XpressBio Co, Thurmont, MD) according to the manufacturer’s instructions [54]. The concentration of the test compounds was 0.1–100 μM, and the percentage of the integrase activity was calculated by dividing the mean value of the test compound with that of the DMSO control.

## Additional files

10.1186/s12977-015-0218-9. Effect of the A1752 on reverse transcriptase activity in vitro. The percentage inhibition of in vitro RT activity by the compounds indicated at different concentrations is shown. Nevirapine and Etravirine, both HIV-1 non-nucleoside reverse transcriptase inhibitors, were used as positive controls. Data are the mean ± SEM of three separate experiments.
10.1186/s12977-015-0218-9 *In vitro* assay of A1752′s effect on HIV-1 integrase activity. *In vitro* integrase assays were performed with the compounds indicated at concentrations ranging from 0.1–100 μM. Raltegravir and Elvitegravir were used as positive controls. Data are the mean ± SEM of three separate experiments.
10.1186/s12977-015-0218-9 Analysis of binding kinetics and affinity between NCp7 and A1752.
10.1186/s12977-015-0218-9 Determination of NC-mediated cTAR DNA destabilization. Emission spectra of Rh6G-5′-cTAR-3′-DABCYL (0.1 μM) were measured in the absence or in the presence of NC (1 μM). Excitation wavelength was fixed at 520 nm and emission wavelength was scanned at 450 to 650 nm. One representative of two independent experiments was shown.
10.1186/s12977-015-0218-9 Inhibition of HIV-1 gRNA packaging in a high concentration of A1752. MT4 cells were infected with treatment of the inhibitors indicated. The viral genomic RNA was isolated from concentrated viral supernatant followed by northern blot analysis using Gag-specific probes.
10.1186/s12977-015-0218-9 Induction of hyper-stable core of HIV-1 by A1752. MT4 cells were infected with HIV-1 NL4-3/EGFP virus together with the A1752 and Tenofovir treatment at an increasing concentration. The released virus particles were permeabilized with melittin at indicated amounts, followed by incubation at 37 °C for 30 min. The pellet and supernatant fraction were analyzed using western blot assays with anti-CA antibodies.
