# Supplementary material for: Identification and characterization of a new type of inhibitor against the human immunodeficiency virus type-1 nucleocapsid protein
Source: Retrovirology. 2015 Nov 6;12:90. doi: 10.1186/s12977-015-0218-9 (PMC4636002; doi:10.1186/s12977-015-0218-9)
Supplement: Supplementary file 1 — 10.1186/s12977-015-0218-9. Effect of the A1752 on reverse transcriptase activity in vitro. The percentage inhibition of in vitro RT activity by the compounds indicated at different concentrations is shown. Nevirapine and Etravirine, both HIV-1 non-nucleoside reverse transcriptase inhibitors, were used as positive controls. Data are the mean ± SEM of three separate experiments. [file 12977_2015_218_MOESM1_ESM.pdf]

Additional file 1.

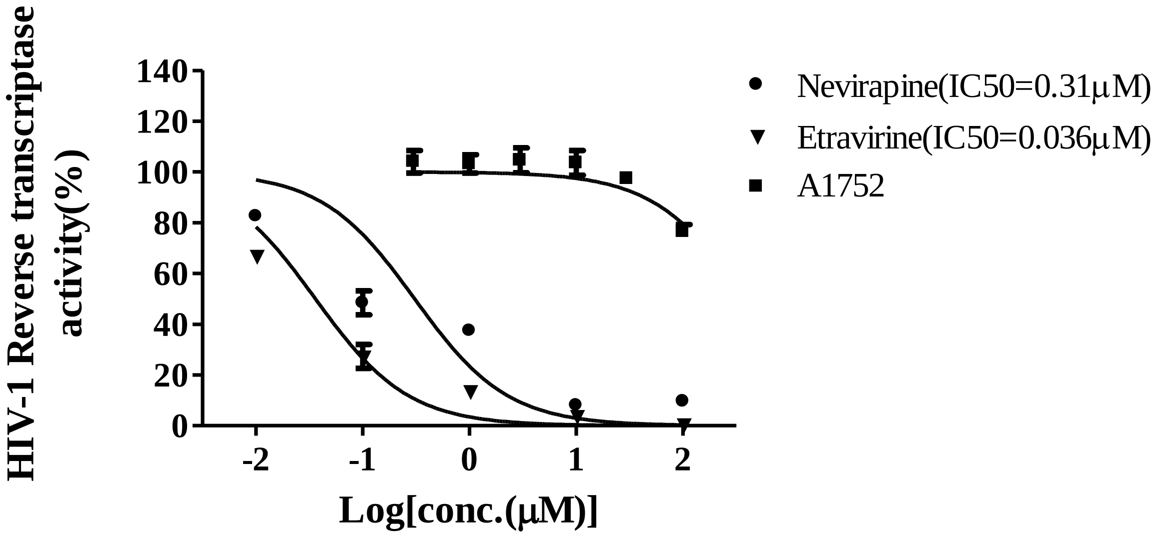

Additional file 1: Figure S1. Effect of the A1752 on reverse transcriptase activity *in vitro*.

The percentage inhibition of *in vitro* RT activity by the compounds indicated at different concentrations is shown. Nevirapine and Etravirine, both HIV-1 non-nucleoside reverse transcriptase inhibitors, were used as positive controls. Data are the mean ± SEM of three separate experiments.
