# Supplementary material for: Identification and characterization of a new type of inhibitor against the human immunodeficiency virus type-1 nucleocapsid protein
Source: Retrovirology. 2015 Nov 6;12:90. doi: 10.1186/s12977-015-0218-9 (PMC4636002; doi:10.1186/s12977-015-0218-9)
Supplement: Supplementary file 2 — 10.1186/s12977-015-0218-9 In vitro assay of A1752′s effect on HIV-1 integrase activity. In vitro integrase assays were performed with the compounds indicated at concentrations ranging from 0.1–100 μM. Raltegravir and Elvitegravir were used as positive controls. Data are the mean ± SEM of three separate experiments. [file 12977_2015_218_MOESM2_ESM.pdf]

Additional file 2.

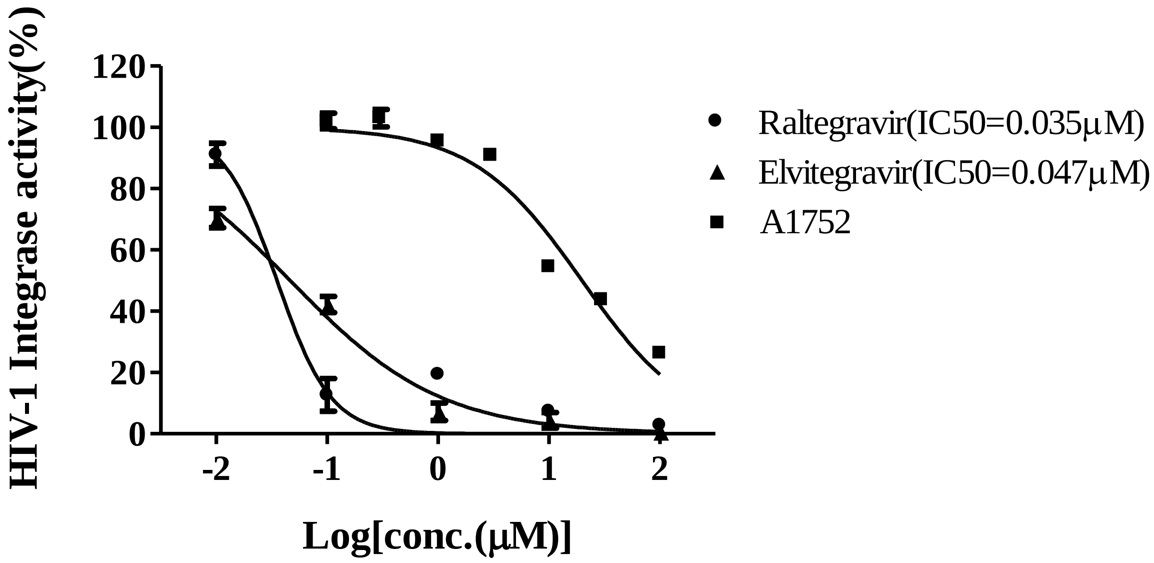

Additional file 2: Figure S2. *In vitro* assay of A1752's effect on HIV-1 integrase activity.

In vitro integrase assays were performed with the compounds indicated at concentrations ranging from 0.1–100 μM. Raltegravir and Elvitegravir were used as positive controls. Data are the mean ± SEM of three separate experiments.
