# Supplementary material for: Identification and characterization of a new type of inhibitor against the human immunodeficiency virus type-1 nucleocapsid protein
Source: Retrovirology. 2015 Nov 6;12:90. doi: 10.1186/s12977-015-0218-9 (PMC4636002; doi:10.1186/s12977-015-0218-9)
Supplement: Supplementary file 3 — 10.1186/s12977-015-0218-9 Analysis of binding kinetics and affinity between NCp7 and A1752. [file 12977_2015_218_MOESM3_ESM.pdf]

Additional file 3: Table S1. Analysis of binding kinetics and affinity between NCp7 and A1752.

k_a (1/Ms)	k_d (1/s)	K_D (M)	R_{max} (RU)	t_c	Chi^2 (RU^2)
434.876	9.42E-06	2.17E-08	53.60576	1.02E+08	4.724781

k_a: Association rate constant, k_d: Dissociation rate constant, K_D: Equilibrium dissociation constant (k_d/k_a), R_{max}: Binding capacity of surfaces, t_C: The flow rate-independent component of the mass transfer constant, Chi^2: Total average deviation between the experimental data and the fitted curves.
