# Supplementary material for: Identification and characterization of a new type of inhibitor against the human immunodeficiency virus type-1 nucleocapsid protein
Source: Retrovirology. 2015 Nov 6;12:90. doi: 10.1186/s12977-015-0218-9 (PMC4636002; doi:10.1186/s12977-015-0218-9)
Supplement: Supplementary file 4 — 10.1186/s12977-015-0218-9 Determination of NC-mediated cTAR DNA destabilization. Emission spectra of Rh6G-5′-cTAR-3′-DABCYL (0.1 μM) were measured in the absence or in the presence of NC (1 μM). Excitation wavelength was fixed at 520 nm and emission wavelength was scanned at 450 to 650 nm. One representative of two independent experiments was shown. [file 12977_2015_218_MOESM4_ESM.pdf]

Additional file 4.

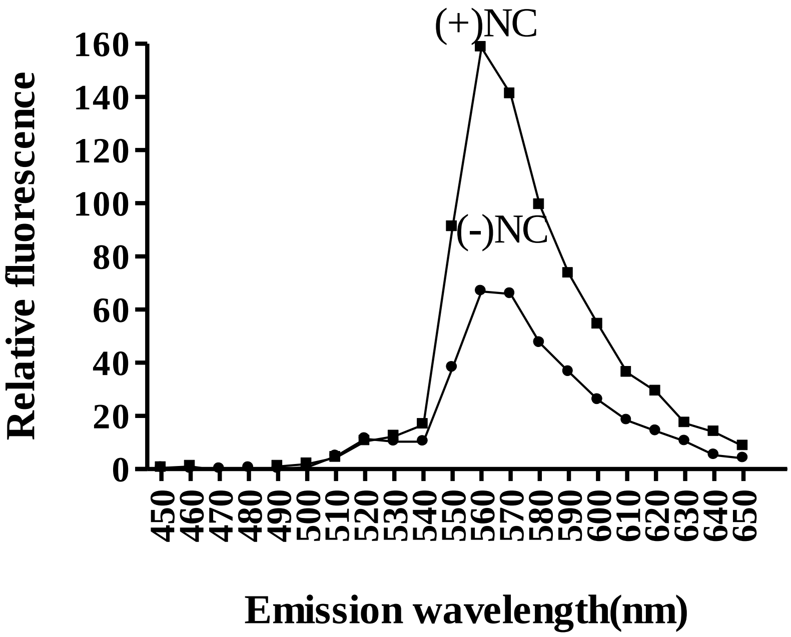

Additional file 4: Figure S3. Determination of NC-mediated cTAR DNA destabilization.

Emission spectra of Rh6G-5'-cTAR-3'-DABCYL (0.1 μM) were measured in the absence or in the presence of NC (1 μM). Excitation wavelength was fixed at 520 nm and emission wavelength was scanned at 450 to 650 nm. Two independent experiments were represented.
