# Supplementary material for: Identification and characterization of a new type of inhibitor against the human immunodeficiency virus type-1 nucleocapsid protein
Source: Retrovirology. 2015 Nov 6;12:90. doi: 10.1186/s12977-015-0218-9 (PMC4636002; doi:10.1186/s12977-015-0218-9)
Supplement: Supplementary file 5 — 10.1186/s12977-015-0218-9 Inhibition of HIV-1 gRNA packaging in a high concentration of A1752. MT4 cells were infected with treatment of the inhibitors indicated. The viral genomic RNA was isolated from concentrated viral supernatant followed by northern blot analysis using Gag-specific probes. [file 12977_2015_218_MOESM5_ESM.pdf]

Additional file 5.

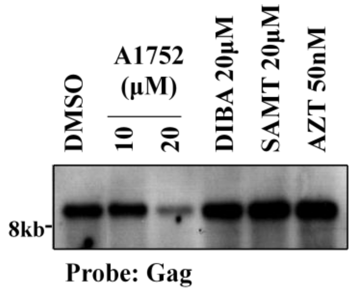

Additional file 5: Figure S4. Inhibition of HIV-1 gRNA packaging at high concentration of A1752.

MT4 cells were infected with treatment of indicated inhibitors. The viral RNA was isolated from concentrated viral supernatant, followed by northern blot analysis using Gag-specific probes.
