# Supplementary material for: Identification and characterization of a new type of inhibitor against the human immunodeficiency virus type-1 nucleocapsid protein
Source: Retrovirology. 2015 Nov 6;12:90. doi: 10.1186/s12977-015-0218-9 (PMC4636002; doi:10.1186/s12977-015-0218-9)
Supplement: Supplementary file 6 — 10.1186/s12977-015-0218-9 Induction of hyper-stable core of HIV-1 by A1752. MT4 cells were infected with HIV-1 NL4-3/EGFP virus together with the A1752 and Tenofovir treatment at an increasing concentration. The released virus particles were permeabilized with melittin at indicated amounts, followed by incubation at 37 °C for 30 min. The pellet and supernatant fraction were analyzed using western blot assays with anti-CA antibodies. [file 12977_2015_218_MOESM6_ESM.pdf]

Additional file 6.

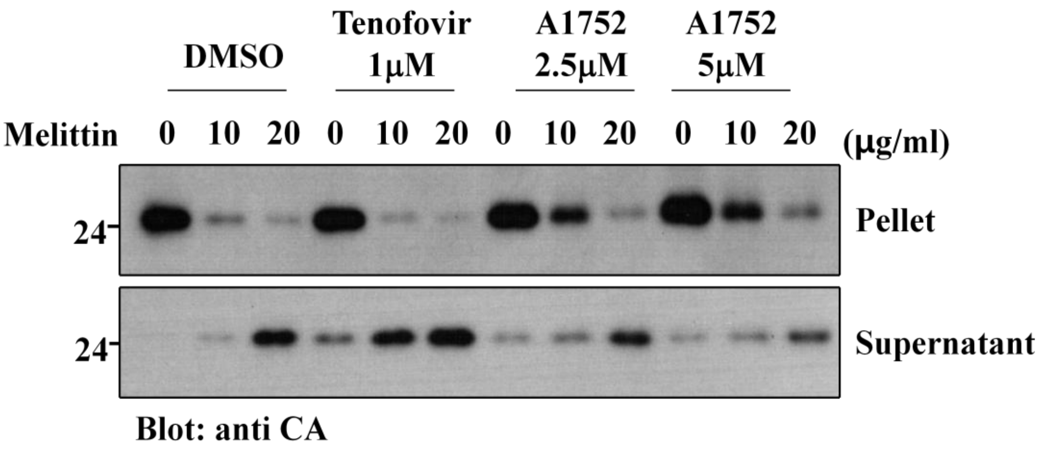

Additional file 6: Figure S5. Induction of hyper-stable core of HIV-1 by A1752.

MT4 cells were infected with HIV-1 NL4-3/EGFP virus together with the A1752 and Tenofovir treatment at an increasing concentration. The released virus particles were permeabilized with melittin at indicated amounts, followed by incubation at 37℃ for 30 min. The pellet and supernatant fraction were analyzed using western blot assays with anti-CA antibodies.
